# Endemic and Imported Measles Virus–Associated Outbreaks among Adults, Beijing, China, 2013

**DOI:** 10.3201/eid2103.140646

**Published:** 2015-03

**Authors:** Meng Chen, Yan Zhang, Fang Huang, Huiling Wang, Donglei Liu, Juan Li, Lance Rodewald, Jiang Wu, Ying Deng, Wenbo Xu

**Affiliations:** Beijing Center for Diseases Prevention and Control, Beijing, China (M. Chen, F. Huang, D. Liu, J. Li, J. Wu, Y. Deng);; National Institute for Viral Disease Control and Prevention, Beijing (Y. Zhang, H. Wang, W. Xu);; World Health Organization China Representative Office, Beijing (L. Rodewald)

**Keywords:** adults, measles, outbreak, genotype H1, genotype D8, Beijing, China, viruses, endemic measles, imported measles

## Abstract

In 2013, a resurgence of measles occurred in Beijing, China. The outbreaks occurred among adults and were associated with endemic genotype H1 and imported genotype D8 viruses. Migrant workers were disproportionately represented in the outbreaks; thus, vaccinating such workers against measles may be an effective strategy toward the elimination of this disease.

All 6 World Health Organization (WHO) regions have set goals to eliminate measles (*1*,*2*). In China, a nationwide measles supplementary immunization activity was conducted in 2010, and the incidence of measles in mainland China subsequently reached its lowest reported level in 2012 (6,183 cases, 4.6 cases/million total population). However, in 2013, a nationwide resurgence of measles occurred primarily among young, unvaccinated children (*3*). In contrast to the nationwide resurgence, the measles resurgence in China’s capital, Beijing, was primarily among adults >15 years of age (65.7% of cases) and occurred in large, clothing wholesale markets.

Beijing has >12.96 million permanent residents and an additional ≈7.73 million floating residents (i.e., internal migrants who move into the city, usually for employment) (*4*). The routine measles vaccination schedule in use in Beijing consists of 3 doses of measles-containing vaccine; the first dose is administered at 8 months, the second at 18 months, and the third at 6 years of age. Also, since 2006, an additional dose has been administered to college students who move to Beijing to study (*5*). In this study, we used genotype analysis to describe the measles outbreaks among adults in Beijing, and we suggest an immunization strategy to help prevent similar outbreaks in the future.

## The Study

In early 2013, a resurgence of measles in Beijing was reported to the China Information System for Disease Control and Prevention, Chinese Center for Disease Control and Prevention (Beijing); by the end of the year, a total of 1,233 suspected cases had been reported and investigated. Serum and throat swab samples were collected from 97.3% and 96.8% of the suspected case-patients, respectively. The samples were tested for measles IgM by using the VIRION SERION ELISA Measles Virus IgM test (Virion/Serion, Wurzburg, Germany) or for measles virus genes by using a real-time reverse transcription PCR kit (Jiangsu Bioperfectus Technologies, Jiangsu, China). Of the 1,233 samples, 558 were positive, and 5 additional cases were confirmed by epidemiologic linkage. Thus, a total of 563 measles cases in Beijing were confirmed in 2013; this number represents a 6-fold increase from the number of cases in 2012.

The number of reported cases was highest during March–May. Most cases occurred among a floating population of adult migrant workers and permanent adult residents and their children (Figure); 67.3% of the cases in adults were in migrant workers. Reports of cases increased shortly after the national holidays associated with the spring festival, during which many persons travel to visit relatives.

**Figure:**
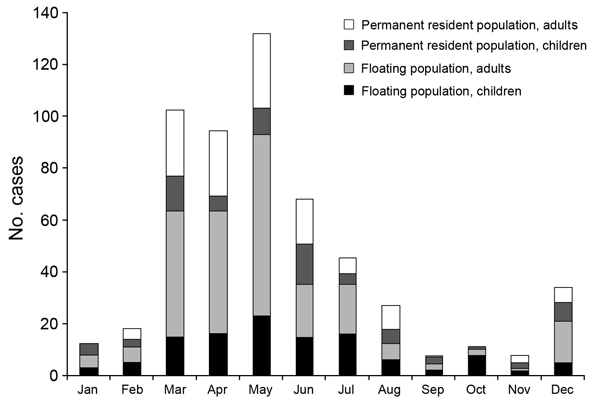
Number of measles cases among persons by residency status, age, and month of infection onset, Beijing, China, 2013. Floating populations represent internal migrants who move to an area temporarily, usually for employment (e.g., migrant workers).

Among the persons with confirmed measles, 22.8% (128) were <8 months of age, 11.5% (65) were 8 months–14 years of age, and 65.7% (370) were >15 years of age and defined as adult patients. The median age of the adult patients was 23.5 years (range 15.0–70.0 years). Vaccination history was unknown for 87.6% (324/370) of the adults. Among the 65 patients in the age group targeted for measles vaccination, 36.9% (24) had not been vaccinated because of contraindications.

We used real-time reverse transcription PCR–positive samples to try to isolate and genotype virus from 468 of the 563 positive samples. Genomic sequencing and phylogenetic analyses were based on N450 nucleotide sequences, as previously described (*6*–*8*). Measles genotype was determined for 45.6% (257/563) of the cases reported from 14 of 16 districts or counties. Among those 257 cases, 84.0% (216) were caused by endemic genotype H1 viruses and 15.2% (39) and 0.78% (2), respectively, were caused by imported genotype D8 and D9 viruses. All 14 districts had confirmed measles cases caused by genotype H1 viruses, and 6 and 2 districts, respectively, had cases caused by genotype D8 and D9 viruses. Of the 257 measles cases with genotype information, 61.6% (133) of those caused by genotype H1 and 97.4% (38) of those caused by genotype D8 were in adults (Table).

**Table:** Age distribution of measles case-patients for whom measles virus genotype information was available, Beijing, China, 2013

Age group	No. (%) genotype		
	H1	D8	D9
0–7 mo	31 (14.3)	1 (2.6)	1 (50.0)
8 mo–14 y	52 (24.1)	0	0
>15 y	133 (61.6)	38 (97.4)	1 (50.0)

Measles viruses within a transmission chain had identical or nearly identical N450 sequences (*9*). Phylogenetic analysis showed that 216 genotype H1 viruses were associated with 30 different chains of transmission (Technical Appendix Figure, panels A, B), and 39 genotype D8 viruses were associated a single chain of transmission, although 1 virus differed by only 1 nt (Technical Appendix Figure, panels A, C). Nucleotide sequences of 43 representative viruses were deposited in GenBank (accession nos. KJ556851–93). A search of the World Health Organization’s Measles Nucleotide Surveillance (MeaNS) database (*10*) showed that genomic sequences of genotype D8 viruses from the outbreak shared 99.8%–100% nucleotide identity with genomic sequences of strains from measles patients in Russia, France, Canada, Thailand, Denmark, Germany, and other locations.

Genotype D8 measles viruses were associated with at least 2 outbreaks in different large, wholesale clothing markets. The outbreaks occurred during March–July 2013 and were almost completely confined to adults; only 1 child was infected, possibly because of high coverage of measles-containing vaccine among Beijing children. We were unable to identify the source of the infections. Phylogenetic analysis suggested that the genotype D8 virus might have been imported to Beijing from Europe, America, or another location (*10*–*13*) and subsequently spread beyond Beijing by virus transmission from infected adults (data not shown). For at least 21 years, genotype H1 measles viruses have been the only endemic measles circulating in China (*6*–*8*); measles cases caused by all other genotypes have been associated with imported viruses (*7*).

## Conclusions

Our findings show that transmission of measles virus among adults contributed to a resurgence of measles in Beijing during 2013. Nonendemic genotype D8 measles viruses were associated with at least 2 outbreaks in different wholesale clothing markets during March–July, 2013. Many persons from domestic and international areas visit wholesale markets every day; thus, such markets are high-risk settings for the transmission and importation of measles viruses (*14*).

Because migrant workers were disproportionately affected in the Beijing outbreaks and because their work settings have high measles transmission potential, we support an outreach strategy to prevent measles among this floating population. These workers usually live with their families and register with the local authorities for government services. Thus, we suggest that the offer of measles vaccine to workers as they register to live and work in the commodity markets might be a reasonable strategy to prevent future measles outbreaks. Serologic surveys can be used to refine such a strategy by assessing immunity within the target population.

The foundation strategy for eliminating measles globally is based on the timely vaccination of young children with 2 valid doses of measles-containing vaccine. However, laboratory-supported surveillance analysis and outbreak investigations are critical to the identification of gaps in immunity among older age groups, which may need to be filled, and to the identification of strategies to prevent similar outbreaks. The fact that more than a third of infected children in the vaccine-targeted age group were unvaccinated because of vaccination contraindications suggests that an evaluation is needed to ensure the use of valid contraindications only.

It is difficult to identify narrow, age-based risk groups to target for vaccination when a high proportion of adults are unvaccinated and may still be susceptible to measles. Unselective vaccination of millions of adults, based solely on their age, is unlikely to be feasible in China. Additional risk factors for measles need to be identified to develop more feasible immunization strategies.

Technical Appendix Phylogenetic analysis comparing genomic sequences of measles virus isolates from persons infected during a 2013 outbreak in Beijing, China, with genomic sequences of World Health Organization (WHO) reference sequences.
