# Supplementary material for: Endemic and Imported Measles Virus–Associated Outbreaks among Adults, Beijing, China, 2013
Source: Emerg Infect Dis. 2015 Mar;21(3):477–9. doi: 10.3201/eid2103.140646 (PMC4344261; doi:10.3201/eid2103.140646)
Supplement: Technical Appendix — Phylogenetic analysis comparing genomic sequences of measles virus isolates from persons infected during a 2013 outbreak in Beijing, China, with genomic sequences of World Health Organization (WHO) reference sequences. [file 14-0646-Techapp-s1.pdf]

Article DOI: http://dx.doi.org/10.3201/eid2103.140646

Endemic and Imported Measles Virus–Associated Outbreaks among Adults, Beijing, China, 2013

Technical Appendix

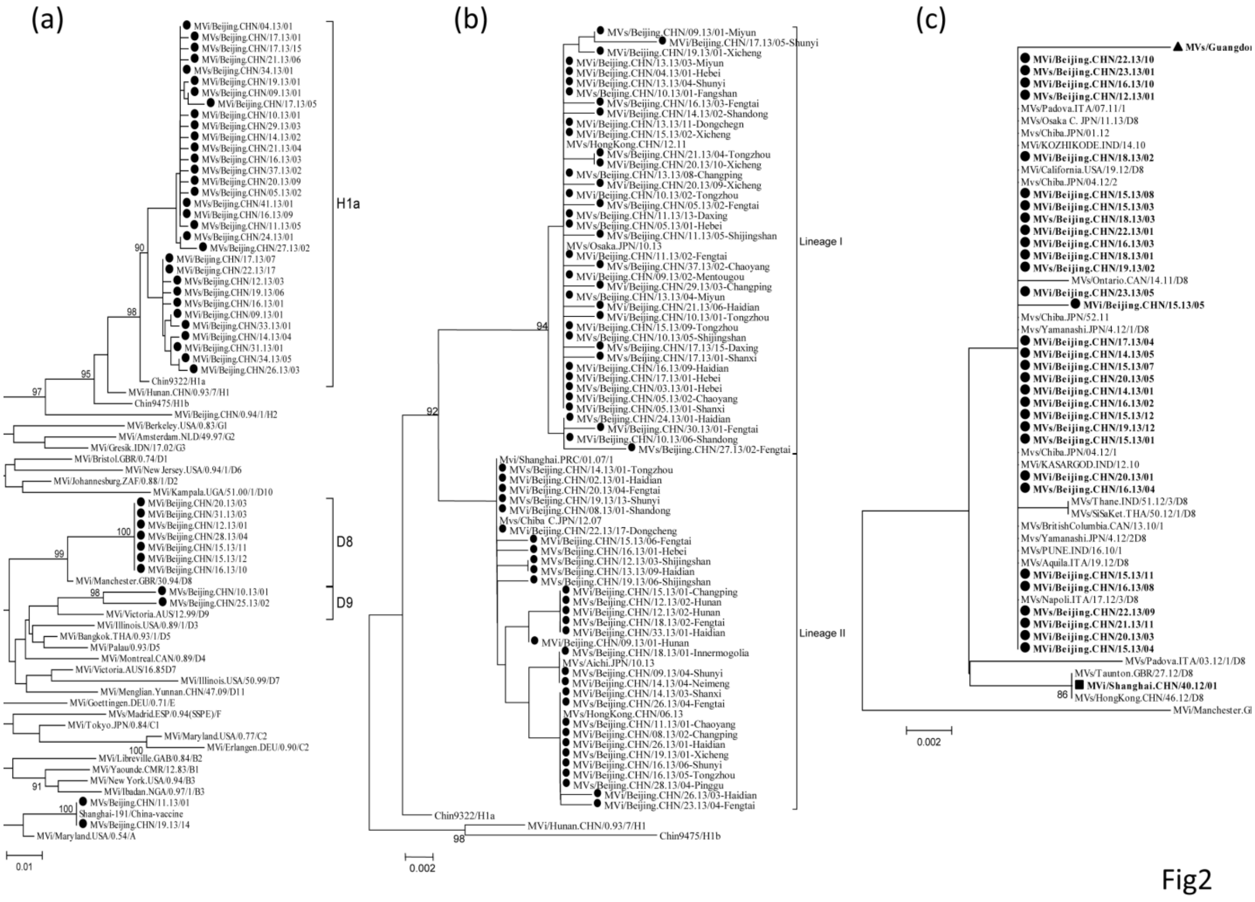

Technical Appendix Figure. Phylogenetic analysis comparing genomic sequences of measles virus isolates from persons infected during a 2013 outbreak in Beijing, China, with genomic sequences of World Health Organization (WHO) reference sequences. MEGA 5.03 (http://megasoftware.net/mega.php) and the neighbor-joining method (1,000 bootstraps) were used for the analysis. The trees are based on

the WHO standard sequence window within the N gene. Measles strains were named according to WHO nomenclature (i.e., MVi/City of isolation.country/epidemiologic week.year of isolation/isolate number-province) (*1*). Dots indicate the Beijing sequences in this study. A): Phylogenetic tree of representative Beijing strains from this study compared with the WHO reference sequences for each genotype. B) Phylogenetic tree of all genotype H1 strains in this study compared with the WHO reference sequences. C) Phylogenetic tree of all genotype D8 strains in this study compared with the WHO reference sequences. Genomic sequences of genotype D8 strains from Russia, France, Canada, Thailand, Denmark, and Germany are not shown because they were not available in GenBank or the MeaNS database (Measles Nucleotide Surveillance database, http://www.who-measles.org). AUS, Australia; CAN, Canada; CHN, China; CMR, Cameroon; DEU, Germany; ESP, Spain; GAB, Gabon; GBR, Great Britain; IDN, Indonesia; IND, India; ITA, Italy; JPN, Japan; MeV, measles virus; MVi, measles virus isolate; NGA, Nigeria; NLD, the Netherlands; PRC, People's Republic of China; THA, Thailand; USA, United States of America; ZAF, South Africa. Scale bars represent percentage nucleotide distance.

Reference

1. World Health Organization. Update of the nomenclature for describing the genetic characteristics of wild-type measles viruses: new genotypes and reference strains. Wkly Epidemiol Rec. 2003;78:229–32. PubMed
